# Non-response to a life course socioeconomic position indicator in surveillance: comparison of telephone and face-to-face modes

**DOI:** 10.1186/1471-2288-8-54

**Published:** 2008-08-13

**Authors:** Catherine R Chittleborough, Anne W Taylor, Fran E Baum, Janet E Hiller

**Affiliations:** 1 Discipline of Public Health, School of Population Health and Clinical Practice, Level 9, 10 Pulteney Street, Mail Drop 207, University of Adelaide, South Australia 5005, Australia; 2 Population Research and Outcome Studies Unit, Department of Health, Adelaide, South Australia, Australia; 3 Department of Public Health, Flinders University of South Australia, Bedford Park, South Australia, Australia

## Abstract

**Background:**

Measurement of socioeconomic position (SEP) over the life course in population health surveillance systems is important for examining differences in health and illness between different population groups and for monitoring the impact of policies and interventions aimed at reducing health inequities and intergenerational disadvantage over time. While face-to-face surveys are considered the gold standard of interviewing techniques, computer-assisted telephone interviewing is often preferred for cost and convenience. This study compared recall of parents' highest level of education in telephone and face-to-face surveys.

**Methods:**

Questions about father's and mother's highest education level were included in two representative population health surveys of South Australians aged 18 years and over in Spring 2004. A random sample selected from the electronic white pages (EWP) responded to a computer-assisted telephone interview (n = 2999), and a multistage clustered area sample responded to a face-to-face interview (n = 2893). A subsample of respondents in the face-to-face sample who owned a telephone that was listed in the EWP (n = 2206) was also compared to the telephone interview sample.

**Results:**

The proportion of respondents who provided information about their father's and mother's highest education level was significantly higher in the face-to-face interview (86.3% and 87.8%, respectively) than in the telephone interview (80.4% and 79.9%, respectively). Recall was also significantly higher in the subsample of respondents in the face-to-face interview who had a telephone that was listed in the EWP. Those with missing data for parents' education were more likely to be socioeconomically disadvantaged regardless of the survey mode.

**Conclusion:**

While face-to-face interviewing obtained higher item response rates for questions about parents' education, survey mode did not appear to influence the factors associated with having missing data on father's or mother's highest education level.

## Background

Telephone and face-to-face modalities are commonly used in surveillance systems to obtain information about the health and socioeconomic position (SEP) of the population over time. Face-to-face interviews have traditionally been considered the gold standard [1,2] because of their ability to obtain high unit and item response rates [3,4] and valid data [5]. Detailed information can also be captured in face-to-face interviews because longer interviews can be conducted [3] and support can be provided by interviewers as respondents observe non-verbal expressions [6]. Telephone survey methods are often preferred over face-to-face methods because of the convenience of computer-assisted telephone interviewing (CATI) and the increasing costs of conducting face-to-face surveys [7]. Telephone surveys are quicker, less costly, less intrusive to respondents, have comparable response rates, and are easier to administer and maintain quality control with monitoring of interviewers than face-to-face surveys [3,5,8,9]. Agreement between telephone and face-to-face surveys in the prevalence of health conditions and risk factors has also been demonstrated [8,10].

Surveillance systems traditionally measure current SEP among adults, but do not collect information about respondents' early life SEP. SEP refers to both the social and material resources that influence the position people occupy in society [11,12]. There is increasing evidence about the association of SEP over the life course and health in adulthood [13,14]. Many indicators can be used to measure early life SEP [15]. Mother's and father's highest level of education are commonly used indicators of early life SEP because they are considered direct measures of the status-based construct of SEP that remains relatively stable during adulthood. Inclusion of such questions in surveillance systems to obtain information about early life SEP will enable monitoring of the impact of policies designed to alter trajectories based on early life SEP. It will also allow a more nuanced assessment of the role of SEP. For example, the ability to monitor trends in health status between groups with different life course experiences – those who are disadvantaged in early life and remain disadvantaged through adulthood, those who are disadvantaged in early life but advantaged during adulthood (upward mobility), those who experience downward mobility from childhood to adulthood, and those who are advantaged during both early life and adulthood. In particular, surveillance systems could then assess the impact of policies designed to counter the impact of disadvantage.

Surveillance systems are different from longitudinal cohort studies. They do not provide aetiological evidence that low SEP during childhood causes poor health outcomes in adulthood. The trend data they can provide are necessary, however, to monitor the effectiveness of policies and programs, such as those related to education, employment, early childhood development and those aimed at changing health-related behaviours among the population, and specifically whether such policies have a differential effect on groups with different socioeconomic life course experiences. Conducting such trend analyses among specific age cohort strata would also provide insights into the effects of historical programs and policies among the various social mobility groups. In addition, health promotion campaigns targeted at the general population may be more successful among advantaged groups who may be more health literate. But are such campaigns, programs, or policies least successful among those who have experienced cumulative disadvantage over their lifetime? Are they more successful among those who were disadvantaged in early life but are more advantaged in adulthood? Answers to these questions can be provided in a timely and relatively inexpensive way, by well-designed surveillance systems.

To obtain information from respondents about their early life SEP, surveillance systems necessarily rely on retrospectively recalled information. It is unknown whether survey mode influences item non-response to questions related to early life SEP. The aim of this paper is to compare the extent of missing data to questions about parents' highest education level obtained retrospectively from similar telephone and face-to-face representative population surveys.

## Methods

### Telephone survey

In the Health Monitor telephone survey conducted by the South Australian Department of Health in September 2004, households were randomly selected from those in South Australia with a telephone connected and the number listed in the Electronic White Pages (EWP). The person aged 18 years or over who was last to have a birthday was selected for interview. Up to six call backs were made in an attempt to interview them as selected persons were non-replaceable. A letter was sent to each selected household prior to interview, to inform about the purpose of the survey. CATI was used to conduct the interviews. From the eligible sample (n = 4342), 714 refused to participate in the interview, 233 were not contactable after six attempts, 160 were incapacitated, 135 were unavailable for interview, 90 were excluded due to language barriers (interviews were only conducted in English, Greek, Italian and Vietnamese), and eight terminated the interview. An additional three respondents were excluded due to missing data required for weighting purposes. A total of 2999 completed interviews were analysed, a response rate of 69.1%.

### Face-to-face survey

The South Australian Health Omnibus Survey is a systematic, self-weighting, multistage, clustered area sample of the metropolitan area and regional centres with a population greater than 1,000. Hotels, motels, hospitals, nursing homes and other major institutions are excluded. To achieve a sample of 4500 households, 10 households were randomly selected from each of 450 collector districts (CD). A CD is a Census-defined geographical area comprising approximately 200 dwellings. CDs and households were selected using a fixed skip interval from a random starting point. An approach letter introducing the survey was sent to all selected households. Within households, the person aged 15 years or over who was last to have a birthday was selected for interview. Up to six call-backs were made to interview the selected person and there were no replacements for non-respondents. From the eligible sample (n = 4366), 808 refused to participate in the interview, 316 were not contactable after six attempts, 61 were incapacitated, 74 were unavailable for interview, 72 did not speak English, and one terminated the interview. An additional 49 households were unable to be accessed because of locked gates or because the interviewer considered it unsafe to enter. A total of 2985 interviews were completed in Spring 2004, a response rate of 68.4%. The sample size of the face-to-face survey was reduced to n = 2893 after respondents aged 15 to 17 years were excluded.

A subsample of the face-to-face survey consisting of those aged 18 years and over who owned a telephone that was listed in the EWP (n = 2206, 76.3%) was also used to compare with the telephone survey. Use of this EWP subsample enabled a closer match with the telephone sample to better compare the effect of mode rather than differences in survey sample characteristics.

### Measures of SEP and health

Father and mother's highest level of education were included in both the telephone and face-to-face survey. Responses categorised as "don't know" were classed as missing. No respondents were coded as having refused to answer the questions about parents' highest level of education.

The respondent's main occupation, employment status, household income, and highest level of education were included as measures of current SEP. Respondents were asked what kind of work they had done for most of their life. Occupations were coded according to the Australian Standard Classification of Occupations (ASCO), which groups occupations according to level of education, knowledge, responsibility and on the job training and experience required [16]. Occupations were coded into five groups: 1) Manager, administrator, professional, 2) Associate professional, 3) Tradesperson, advanced clerical or service, 4) Intermediate clerical/service/sales/production/transport, and 5) Elementary clerical/sales/service/labourer. Respondents whose main occupation was home duties or student and those who had never worked or did not state their occupation were combined into a sixth category. Additional sociodemographic factors collected included age, sex, area of residence, marital status, and country of birth.

### Data analysis

Data in each survey were weighted by age group, sex, geographical area, and household size to the Estimated Residential Population [17] aged 18 years and over to account for different probabilities of selection and response rates among different demographic groups, thus ensuring that the sample accurately reflected the South Australian adult population.

Univariate comparisons of the demographic profile and the distribution of the parental education variables by survey mode were made using chi-square tests in SPSS version 13 (SPSS Inc, Chicago, Illinois, USA). To examine the association between having missing data on father's or mother's highest education level and current demographic and socioeconomic variables, relative risks were calculated by log-binomial regression using Stata version 9 (StataCorp, College Station, Texas, USA). The relative risks presented were adjusted for age because increasing age was a confounding factor associated with missing data on the early life SEP variables. Relative risks were estimated using log binomial regression from the generalized linear modeling (GLM) command in Stata. This procedure was used instead of calculating odds ratios using logistic regression because odds ratios can overstate the association when the outcome is common. In this case, the dependent variable of missing data on parents' highest level of education ranged from approximately 12% to 20%. SPSS was not used here as the GLM procedure did not take account of non-integer weights. Differences were reported as significant when p < 0.01 and p < 0.001.

## Results

The demographic profiles of respondents in the telephone survey, the face-to-face survey, and the EWP subsample of the face-to-face survey (those with a telephone listed in the EWP) are compared in Table 1. Compared to telephone survey respondents, those in the face-to-face survey were significantly more likely to be born outside Australia, the United Kingdom, or Ireland, have a trade, apprenticeship, certificate or diploma education level, a gross annual household income of $20,000 or less, state their employment as home duties, and have an occupation of tradesperson or advanced clerical or service. Those in the face-to-face survey were less likely than telephone respondents to have left school at 15 years of age or younger, to be unemployed or unable to work, and to have the highest status occupation. The EWP subsample of the face-to-face survey were more likely than telephone survey respondents to have a certificate or diploma education level, to state their employment as retired or home duties, and to have an occupation of tradesperson or advanced clerical or service, and less likely to be unemployed or unable to work, to have left school at 15 years of age or younger, or to have never been married.

**Table 1:** Demographic profile of respondents by survey mode

	**Telephone (n = 2999)**	**Face-to-face (n = 2893)**	**Face-to-face EWP (n = 2206)**
	% (95% CI)	% (95% CI)	% (95% CI)
**Sex**			
Male	49.0 (47.2–50.8)	49.0 (47.2–50.8)	48.3 (46.2–50.4)
Female	51.0 (49.2–52.8)	51.0 (49.2–52.8)	51.7 (49.6–53.8)
**Age group (years)**			
18 to 24	12.1 (11.0–13.3)	12.0 (10.9–13.3)	10.2 (9.0–11.5)
25 to 34	17.2 (15.9–18.6)	17.2 (15.9–18.7)	15.4 (13.9–16.9)
35 to 44	19.3 (17.9–20.8)	19.3 (17.9–20.8)	18.7 (17.1–20.4)
45 to 54	18.2 (16.8–19.6)	18.2 (16.8–19.6)	17.9 (16.4–19.6)
55 to 64	13.9 (12.7–15.2)	13.9 (12.7–15.2)	14.9 (13.5–16.5)
65 to 74	9.8 (8.8–10.9)	9.8 (8.8–10.9)	11.7 (10.4–13.1)
75+	9.5 (8.5–10.6)	9.5 (8.5–10.6)	11.2 (10.0–12.6)
**Country of birth**			
Australia	77.9 (76.4–79.3)	74.8 (73.2–76.3)*	75.7 (73.9–77.4)
UK/Ireland	11.1 (10.1–12.3)	11.5 (10.4–12.7)	11.6 (10.3–13.0)
Other	11.0 (9.9–12.1)	13.7 (12.5–15.0)*	12.7 (11.4–14.1)
**Marital status**			
Married/de facto	67.7 (66.0–69.3)	66.1 (64.4–67.8)	69.4 (67.4–71.3)
Separated/divorced/widowed	13.4 (12.2–14.7)	15.3 (14.0–16.7)	14.5 (13.1–16.1)
Never married	18.9 (17.5–20.3)	18.5 (17.2–20.0)	16.0 (14.6–17.6)*
**Area of residence**			
Metropolitan	74.0 (72.4–75.6)	72.8 (71.2–74.4)	72.0 (70.1–73.8)
Country	26.0 (24.4–27.6)	27.2 (25.6–28.8)	28.0 (26.2–29.9)
**Highest education level**			
Still at school	1.6 (1.2–2.1)	0.9 (0.6–1.3)	0.9 (0.6–1.4)
Left school at age 15 or younger	16.1 (14.9–17.5)	12.2 (11.1–13.5)**	12.9 (11.6–14.4)*
Left school after age 15	34.0 (32.3–35.7)	32.0 (30.3–33.7)	31.3 (29.4–33.3)
Trade/Apprenticeship	12.2 (11.0–13.4)	14.6 (13.3–15.9)*	14.5 (13.1–16.0)
Certificate/Diploma	18.3 (16.9–19.7)	23.9 (22.4–25.5)**	24.5 (22.7–26.3)**
Bachelor degree or higher	17.8 (16.5–19.2)	16.4 (15.1–17.8)	15.9 (14.4–17.5)
**Gross annual household income**			
Up to $20,000	15.1 (13.9–16.5)	17.8 (16.4–19.2)*	17.3 (15.8–18.9)
$20,001 to $40,000	18.4 (17.0–19.8)	18.0 (16.7–19.5)	17.2 (15.6–18.8)
$40,001 to $60,000	18.3 (16.9–19.7)	17.8 (16.5–19.3)	17.6 (16.1–19.3)
More than $60,000	37.5 (35.7–39.2)	34.7 (33.0–36.5)	35.6 (33.6–37.6)
Not stated	10.8 (9.7–11.9)	11.6 (10.5–12.8)	12.4 (11.1–13.8)
**Employment status**			
Full time employed	41.8 (40.1–43.6)	40.1 (38.3–41.9)	39.2 (37.1–41.2)
Part time/casual employed	18.6 (17.3–20.1)	17.5 (16.1–18.9)	17.3 (15.7–18.9)
Unemployed/unable to work	5.6 (4.8–6.4)	3.4 (2.8–4.2)**	2.2 (1.7–2.9)**
Home duties	9.1 (8.1–10.2)	11.5 (10.4–12.7)*	11.5 (10.3–12.9)*
Retired	19.4 (18.0–20.9)	20.5 (19.1–22.0)	24.0 (22.2–25.8)**
Student	4.5 (3.8–5.3)	5.1 (4.3–5.9)	4.6 (3.8–5.6)
Not stated	1.0 (0.7–1.4)	1.9 (1.5–2.5)*	1.2 (0.8–1.8)
**Occupation**			
Manager/Administrator/Professional	22.6 (21.1–24.1)	19.6 (18.2–21.1)*	20.6 (19.0–22.4)
Associate professional	8.7 (7.7–9.7)	7.2 (6.3–8.2)	7.1 (6.1–8.2)
Tradesperson/Advanced clerical or service	16.1 (14.8–17.4)	21.2 (19.8–22.7)**	21.6 (19.9–23.3)**
Intermediate clerical/service/sales/production/transport	23.9 (22.4–25.5)	23.9 (22.4–25.5)	23.9 (22.1–25.7)
Elementary clerical, sales, service/labourer	20.2 (18.8–21.7)	19.5 (18.1–20.9)	18.2 (16.7–19.9)
Not stated/Home duties/Student/Never worked	8.5 (7.6–9.6)	8.6 (7.6–9.7)	8.7 (7.6–9.9)

* p < 0.01 ** p < 0.001 Statistically significantly different from telephone survey

On questions about parents' highest level of education, respondents to the telephone survey had lower item response, and a significantly greater proportion of "don't know" responses (Table 2). The proportion of missing data for father's education ranged from 13.7% in the face-to-face survey to 19.6% in the telephone survey. Similarly, for mother's education, the proportion of missing data was 12.2% among respondents in the face-to-face survey and 20.1% among telephone survey respondents.

**Table 2:** Distribution of parents' highest education level by survey mode

	**Telephone**		**Face-to-face**		**Face-to-face EWP**	
	**(n = 2999)**		**(n = 2893)**		**(n = 2206)**	
	%	(95% CI)	%	(95% CI)	%	(95% CI)
**Father's education**						
Completed primary school	19.7	(18.4–21.2)	22.4	(21.0–24.0)	23.7	(22.0–25.6)**
Some high school	23.2	(21.8–24.8)	22.5	(21.0–24.1)	22.3	(20.6–24.1)
Completed high school	9.5	(8.5–10.6)	11.8	(10.7–13.0)*	11.2	(9.9–12.6)
Trade/Diploma	17.8	(16.4–19.2)	17.2	(15.9–18.6)	16.5	(15.0–18.1)
University degree or higher	9.8	(8.8–10.9)	12.2	(11.0–13.4)*	11.9	(10.6–13.3)
Other	0.4	(0.2–0.7)	0.2	(0.1–0.5)	0.2	(0.1–0.5)
Don't know	19.6	(18.2–21.1)	13.7	(12.5–15.0)**	14.1	(12.7–15.6)**
**Mother's education**						
Completed primary school	19.8	(18.5–21.3)	24.4	(22.9–26.0)**	25.8	(24.1–27.7)**
Some high school	28.9	(27.3–30.6)	29.8	(28.2–31.5)	29.8	(27.9–31.7)
Completed high school	14.8	(13.5–16.1)	16.1	(14.8–17.5)	15.2	(13.7–16.7)
Trade/Diploma	9.6	(8.6–10.7)	8.8	(7.8–9.9)	8.0	(6.9–9.2)
University degree or higher	6.5	(5.6–7.4)	8.7	(7.7–9.8)**	8.6	(7.6–9.9)*
Other	0.3	(0.2–0.6)	0.1	(0.0–0.3)	0.1	(0.0–0.3)
Don't know	20.1	(18.7–21.6)	12.2	(11.0–13.4)**	12.5	(11.2–14.0)**

* p < 0.01 ** p < 0.001 Statistically significantly different from telephone survey

The associations of current SEP variables with missing data on parents' highest education level were similar for the telephone and face-to-face samples and the EWP subsample. Across each survey mode, respondents with low current SEP were more likely to have missing data on father's or mother's highest education level (Table 3 and Table 4). After adjusting for age, respondents in both the telephone and face-to-face surveys who had a lower education level, lower household income, an occupation level of tradesperson or advanced clerical or service or less, or who stated their employment status as unemployed, unable to work, home duties or retired, were significantly more likely to have missing data for father's highest education level. Similarly, respondents with a lower education level, lower household income, a lower occupation level or who stated their employment status was unemployed, unable to work, or retired, were significantly more likely to have missing data for mother's highest education level. In addition, respondents in the telephone survey who were born in the United Kingdom or Ireland were also more likely to have missing data on father's and mother's highest education level.

**Table 3:** Unadjusted numbers, proportions and relative risks adjusted for current age for variables associated with having missing data for father's highest education level, by survey mode

	**Telephone (n = 2999)**			**Face-to-face (n = 2893)**			**Face-to-face EWP (n = 2206)**		
	Unadjusted		Adjusted	Unadjusted		Adjusted	Unadjusted		Adjusted
	n	%	(RR, 95% CI)	n	%	(RR, 95% CI)	n	%	(RR, 95% CI)
**Sex**									
Male	268	18.3	(1.00)	190	13.4	(1.00)	154	14.4	(1.00)
Female	319	20.9	(1.11, 0.96–1.28)	206	14.0	(1.00, 0.82–1.21)	157	13.8	(0.93, 0.74–1.16)
**Country of birth**									
Australia	421	18.0	(1.00)	273	12.6	(1.00)	215	12.9	(1.00)
UK/Ireland	103	30.7	(1.33, 1.11–1.58)*	70	21.0	(1.36, 1.06–1.74)	57	22.2	(1.40, 1.07–1.83)
Other	64	19.6	(0.91, 0.72–1.16)	54	13.6	(0.92, 0.68–1.24)	39	14.1	(0.91, 0.64–1.31)
**Marital status**									
Married/de facto	399	19.6	(1.00)	239	12.5	(1.00)	201	13.1	(1.00)
Separated/divorced/widowed	126	31.3	(1.25, 1.07–1.46)*	97	21.9	(1.37, 1.12–1.67)*	68	21.1	(1.24, 0.99–1.56)
Never married	62	11.0	(0.85, 0.62–1.18)	60	11.3	(1.43, 0.99–2.07)	43	12.1	(1.59, 1.03–2.46)
**Area of residence**									
Metropolitan	422	19.0	(1.00)	282	13.4	(1.00)	215	13.6	(1.00)
Country	166	21.3	(1.06, 0.91–1.24)	115	14.6	(1.05, 0.84–1.33)	96	15.5	(1.11, 0.86–1.44)
**Highest education level**									
Bachelor degree or higher	33	6.1	(1.00)	9	2.0	(1.00)	6	1.8	(1.00)
Certificate/Diploma	92	16.7	(2.48, 1.69–3.62)**	68	9.9	(4.73, 2.40–9.34)**	51	9.5	(4.97, 2.24–11.03)**
Trade/Apprenticeship	67	18.5	(2.66, 1.78–3.96)**	51	12.1	(5.51, 2.72–11.14)**	45	14.1	(6.69, 2.93–15.26)**
Left school after age 15	215	21.1	(3.31, 2.33–4.70)**	153	16.6	(7.97, 4.12–15.43)**	116	16.8	(8.64, 3.95–18.87)**
Left school at age 15 or younger	177	36.6	(4.05, 2.81–5.84)**	114	32.4	(11.84, 6.01–23.30)**	93	32.6	(12.67, 5.68–28.28)**
Still at school	3	6.9	(-)	0	0.0	(-)	0	0.0	(-)
**Gross annual household income**									
More than $60,000	126	11.2	(1.00)	50	5.0	(1.00)	39	5.0	(1.00)
$40,001 to $60,000	86	15.7	(1.33, 1.01–1.74)	52	10.1	(2.01, 1.27–3.17)*	44	11.4	(2.23, 1.32–3.77)*
$20,001 to $40,000	123	22.3	(1.58, 1.23–2.04)**	83	16.0	(2.87, 1.90–4.35)**	63	16.5	(2.84, 1.72–4.68)**
Up to $20,000	154	33.9	(2.10, 1.63–2.70)**	134	26.0	(4.16, 2.77–6.26)**	100	26.4	(3.97, 2.43–6.50)**
Not stated	99	30.7	(2.43, 1.86–3.16)**	77	23.0	(4.20, 2.76–6.40)**	65	23.9	(4.27, 2.61–6.99)**
**Employment status**									
Full time employed	158	12.6	(1.00)	89	7.7	(1.00)	63	7.3	(1.00)
Part time/casual employed	96	17.1	(1.35, 1.05–1.74)	38	7.4	(0.95, 0.64–1.42)	29	7.6	(1.01, 0.62–1.63)
Unemployed/unable to work	45	27.1	(2.11, 1.57–2.83)**	21	21.3	(3.08, 1.81–5.26)**	11	23.0	(3.38, 1.70–6.71)*
Home duties	69	25.2	(1.73, 1.31–2.28)**	69	20.8	(2.45, 1.72–3.49)**	52	20.5	(2.42, 1.60–3.73)**
Retired	208	35.7	(1.95, 1.50–2.53)**	147	24.8	(2.40, 1.56–3.69)**	131	24.8	(2.38, 1.41–4.00)*
Student	11	8.1	(0.83, 0.36–1.93)	15	9.9	(1.61, 0.71–3.65)	15	14.4	(2.49, 1.11–5.58)
Not stated	2	5.8	(-)	18	31.8	(3.58, 2.29–5.62)**	10	35.2	(4.01, 2.21–7.26)**
**Occupation**									
Manager/Administrator/Professional	79	11.7	(1.00)	34	6.0	(1.00)	28	6.2	(1.00)
Associate professional	42	16.1	(1.42, 1.01–1.99)	20	9.6	(1.59, 0.95–2.66)	18	11.4	(1.68, 0.98–2.91)
Tradesperson/Advanced clerical or service	109	22.6	(1.86, 1.43–2.41)**	86	14.0	(2.21, 1.49–3.27)**	70	14.7	(2.12, 1.38–3.27)*
Intermediate clerical/service/sales/production/transport	144	20.0	(1.81, 1.41–2.33)**	100	14.4	(2.42, 1.66–3.54)**	76	14.4	(2.27, 1.50–3.44)**
Elementary clerical, sales, service/labourer	157	25.8	(2.40, 1.87–3.08)**	106	18.7	(3.18, 2.17–4.65)**	76	18.9	(2.88, 1.88–4.42)**
Not stated/Home duties/Student/Never worked	57	22.5	(1.86, 1.38–2.52)**	51	20.6	(3.15, 2.06–4.81)**	143	22.5	(3.09, 1.95–4.91)**

* p < 0.01 ** p < 0.001

**Table 4:** Unadjusted numbers, proportions and relative risks adjusted for current age for variables associated with having missing data for mother's highest education level, by survey mode

	**Telephone (n = 2999)**			**Face-to-face (n = 2893)**			**Face-to-face EWP (n = 2206)**		
	Unadjusted		Adjusted	Unadjusted		Adjusted	Unadjusted		Adjusted
	n	%	(RR, 95% CI)	n	%	(RR, 95% CI)	n	%	(RR, 95% CI)
**Sex**									
Male	308	21.0	(1.00)	183	12.9	(1.00)	145	13.6	(1.00)
Female	295	19.2	(0.89, 0.78–1.03)	169	11.5	(0.86, 0.70–1.06)	132	11.6	(0.84, 0.66–1.06)
**Country of birth**									
Australia	431	18.5	(1.00)	239	11.1	(1.00)	187	11.2	(1.00)
UK/Ireland	112	33.9	(1.43, 1.20–1.70)**	63	19.0	(1.33, 1.02–1.73)	51	19.8	(1.36, 1.02–1.82)
Other	59	17.8	(0.83, 0.64–1.07)	49	12.5	(0.93, 0.67–1.27)	39	13.9	(1.00, 0.69–1.44)
**Marital status**									
Married/de facto	418	20.6	(1.00)	213	11.1	(1.00)	176	11.5	(1.00)
Separated/divorced/widowed	118	29.3	(1.14, 0.97–1.33)	94	21.3	(1.47, 1.19–1.82)**	64	20.1	(1.30, 1.02–1.65)
Never married	67	11.9	(0.88, 0.65–1.20)	45	8.4	(1.29, 0.82–2.03)	36	10.2	(1.71, 1.04–2.83)
**Area of residence**									
Metropolitan	426	19.2	(1.00)	244	11.6	(1.00)	185	11.6	(1.00)
Country	176	22.6	(1.12, 0.96–1.30)	108	13.7	(1.13, 0.89–1.44)	91	14.8	(1.22, 0.94–1.59)
**Highest education level**									
Bachelor degree or higher	33	6.2	(1.00)	7	1.5	(1.00)	6	1.6	(1.00)
Certificate/Diploma	87	15.8	(2.37, 1.57–3.56)**	57	8.2	(5.00, 2.29–10.89)**	40	7.4	(4.20, 1.68–10.48)*
Trade/Apprenticeship	78	21.5	(3.11, 2.06–4.70)**	58	13.9	(7.87, 3.62–17.13)**	45	14.0	(6.98, 2.79–17.45)**
Left school after age 15	223	21.8	(3.44, 2.35–5.02)**	129	13.9	(8.55, 4.01–18.24)**	103	14.9	(8.15, 3.32–19.98)**
Left school at age 15 or younger	181	37.4	(4.32, 2.93–6.38)**	101	28.6	(12.54, 5.78–27.20)**	83	29.1	(11.25, 4.53–27.94)**
Still at school	0	0.0	(-)	0	0.0	(-)	0	0.0	(-)
**Gross annual household income**									
More than $60,000	137	12.2	(1.00)	46	4.6	(1.00)	37	4.7	(1.00)
$40,001 to $60,000	90	16.5	(1.27, 0.97–1.67)	50	9.7	(2.05, 1.26–3.34)*	42	10.7	(2.20, 1.26–3.84)*
$20,001 to $40,000	127	23.1	(1.53, 1.20–1.95)*	68	13.0	(2.40, 1.51–3.80)**	46	12.1	(2.02, 1.17–3.49)
Up to $20,000	146	32.1	(1.86, 1.45–2.38)**	119	23.1	(3.64, 2.36–5.61)**	90	23.7	(3.27, 1.97–5.42)**
Not stated	102	31.7	(2.33, 1.80–3.01)**	69	20.5	(3.81, 2.40–6.05)**	62	22.7	(3.93, 2.31–6.68)**
**Employment status**									
Full time employed	178	14.3	(1.00)	88	7.6	(1.00)	63	7.3	(1.00)
Part time/casual employed	101	18.0	(1.25, 0.98–1.59)	35	6.9	(0.90, 0.59–1.37)	26	6.9	(0.92, 0.56–1.53)
Unemployed/unable to work	47	28.2	(1.91, 1.43–2.55)**	19	18.7	(2.83, 1.57–5.08)*	10	21.1	(3.20, 1.53–6.70)*
Home duties	61	22.3	(1.35, 1.03–1.77)	49	14.6	(1.60, 1.09–2.37)	37	14.4	(1.50, 0.94–2.41)
Retired	202	34.7	(1.62, 1.26–2.08)**	138	23.2	(1.82, 1.19–2.77)*	123	23.2	(1.69, 1.02–2.81)
Student	11	7.9	(0.72, 0.31–1.71)	11	7.7	(1.43, 0.59–3.43)	10	10.2	(1.97, 0.77–5.05)
Not stated	4	12.3	(-)	13	23.1	(2.47, 1.48–4.13)*	7	25.7	(2.77, 1.37–5.58)*
**Occupation**									
Manager/Administrator/Professional	89	13.2	(1.00)	21	3.7	(1.00)	18	4.1	(1.00)
Associate professional	43	16.6	(1.26, 0.90–1.77)	24	11.3	(2.98, 1.72–5.18)**	18	11.4	(2.59, 1.41–4.77)*
Tradesperson/Advanced clerical or service	113	23.5	(1.67, 1.30–2.15)**	76	12.4	(3.08, 1.94–4.89)**	59	12.5	(2.75, 1.64–4.58)**
Intermediate clerical/service/sales/production/transport	150	20.9	(1.64, 1.29–2.09)**	90	13.0	(3.51, 2.22–5.53)**	73	13.8	(3.36, 2.02–5.57)**
Elementary clerical, sales, service/labourer	159	26.1	(2.13, 1.68–2.69)**	91	16.1	(4.46, 2.82–7.05)**	66	16.3	(3.90, 2.32–6.55)**
Not stated/Home duties/Student/Never worked	48	19.0	(1.44, 1.06–1.95)	51	20.4	(4.93, 3.03–8.03)**	43	22.2	(4.56, 2.66–7.82)**

* p < 0.01 ** p < 0.001

## Discussion

Item-response to mother's and father's education level was higher in the face-to-face survey than the telephone survey. Current socioeconomic disadvantage, however, was associated with a higher prevalence of missing data on questions about parents' education in both survey modes. Socioeconomic differences in health that are based on retrospective recall of parents' education are therefore likely to be under-estimated because those who are socioeconomically disadvantaged are less likely to report their father's or mother's highest education level.

Highest education level of mother or father are commonly used indicators of early life SEP in both prospective and retrospective studies examining the association between SEP and health over the life course [15]. Previous studies relying on retrospective recall have demonstrated higher item response than obtained in this South Australian context. Only 5% of respondents in the Kuopio Ischaemic Heart Disease Risk Factor Study [18] and 6% of respondents in the Coronary Artery (Disease) Risk Development in (Young) Adults (CARDIA) study [19] had missing data for parental education questions. These studies, however, included questions about parents' education in self-administered and face-to-face surveys, and both examined younger samples than the current study. The CARDIA study asked these questions of participants aged 18 to 30 years and the Kuopio study examined middle-aged Finnish men. In addition, participants in the Kuopio study who reported that they did not know one or both of their parents were excluded from the analyses. There are no known studies that have examined the effect of mode on recall of early life SEP. The authors of the Kuopio study noted that retrospective reports of parental education are not as desirable as objective sources of data, but concluded that there was no reason why respondent recall of parents' education should be subject to recall bias [18]. The current results suggest otherwise, with respondents who were socioeconomically disadvantaged less likely to provide information about their parents' education.

The inclusion of questions about telephone ownership and listing in the EWP in the face-to-face survey allowed comparisons to be made between survey mode, independent of sampling frames. The difference in the proportion of respondents with missing data for parents' education between survey modalities could be explained in two ways. First, sociodemographic differences between the selected telephone and face-to-face samples could have influenced item response. If the telephone and face-to-face respondents differed in terms of individual characteristics that were associated with non-response to questions about parents' education, then the estimates of the proportion with missing data on these indicators may be biased by over or under-representing those with missing data [6]. There were few differences, however, between respondents in the telephone survey and those in the face-to-face survey who owned a telephone that was listed in the EWP. Furthermore, these sample characteristic variations are unlikely to have resulted in differences of the magnitude that were observed in the item non-response for parents' education.

Second, differences in interview techniques may also influence responses. For example, while there were no differences in question wording, show cards of the response categories were used in the face-to-face survey. This visual stimulus may have helped improve response rates to questions [1,6]. The "don't know" response option was not offered to respondents in either mode. Responses were only coded as "don't know" if the respondent specifically reported it as their answer.

Face-to-face interviewers may be able to obtain a better rapport with respondents than telephone interviewers [5] and there is also often more time pressure in telephone interviews, which is likely to affect recall [6]. There may also be more opportunities during face-to-face interviews for respondents to interact with other members in the household, or to refer to documentation [6], which may have contributed to the higher item response rates for parents' education in the face-to-face survey.

There can be primacy and recency effects in category order, whereby respondents in face-to-face interviews tend to choose the first category (primacy), and telephone interview respondents choose the last category (most recent) [6]. While primacy effects could have resulted in greater proportions of respondents in the face-to-face interviews selecting the first category of parents' highest education level (completed primary school), these respondents also had a greater proportion of responses in the last category (university degree or higher).

Interview mode differences may be due to variations in interviewer effects and resulting socially desirable responses [7]. While face-to-face interviews are associated with reduced concerns about the legitimacy of interviewers, telephone interviews are associated with greater perceptions of anonymity and tend to result in less socially desirable responses [7] because the expressions and body language of the interviewer are more apparent in face-to-face interviews and may influence answers given [6]. There was no evidence in this study, however, that less socially desirable responses were gained through the anonymity of the telephone survey. In fact, the proportion who reported that their mother or father had a primary school education was higher in the face-to-face survey.

A limitation of this study is that the only indicators of early life SEP available in both the telephone and face-to-face survey were mother's and father's highest education level. Education is a valid measure of SEP [15], and using parents' education as an indicator of early life SEP would still be appropriate in prospective studies that can use more objective measures. Other indicators of early life SEP, such as parents' main occupation, or financial situation during childhood, may perform better than parents' education in terms of response and may therefore be more suitable for inclusion in surveillance systems that rely on retrospective recall. There are also factors, other than item response rates, to consider when deciding on a mode to monitor the association between SEP and health using a life course approach. These include costs and resources available to conduct such surveys, and the characteristics of the target population. Undercoverage of the target population in telephone-based sampling, for example, may be a more serious issue to consider in certain regions than item non-response.

In addition, this study does not provide information on the accuracy of recall of parents' highest level of education. While the face-to-face survey resulted in higher item-response to questions about parents' highest level of education, there is no way to compare the validity of these questions across the different survey modes.

While mode effects were observed in the proportion of respondents who answered questions about their parents' education, there were no differences between the telephone and face-to-face surveys in the demographic and socioeconomic variables associated with having missing data on the indicators of early life SEP. Respondents who were more socioeconomically disadvantaged were more likely to have missing data on mother's and father's highest education level, irrespective of survey mode.

## Conclusion

The face-to-face survey resulted in higher item response to retrospective questions about parents' highest level of education than the telephone survey. This study suggests that parents' highest education level is unlikely to be the most suitable indicator of early life SEP in surveillance systems that rely on retrospective recall because of the high non-response associated with these items, regardless of the survey mode. In addition, current socioeconomic disadvantage was associated with missing data for parents' education within each survey mode. Comparing other indicators of early life SEP, such as parents' occupation, or financial situation during childhood, across different survey modes may determine indicators that are more suitable for use in surveillance systems.

## List of Abbreviations

CATI: computer-assisted telephone interviewing; EWP: electronic white pages; SEP: socioeconomic position.

## Competing interests

The authors declare that they have no competing interests.

## Authors' contributions

CRC was responsible for the conception and design of the study, acquisition of the data, analysis and interpretation of the data, and drafting and critically revising the manuscript for important intellectual content. AWT was involved in acquisition of the data, and critically revising the manuscript for important intellectual content. FEB and JEH contributed to the conception and design of the study, interpreting the data, and critically revising the manuscript for important intellectual content. All authors have given approval of the manuscript version to be published.

## Pre-publication history

The pre-publication history for this paper can be accessed here:


